# Tracking Proteins Secreted by Bacteria: What's in the Toolbox?

**DOI:** 10.3389/fcimb.2017.00221

**Published:** 2017-05-31

**Authors:** Benoit Maffei, Olivera Francetic, Agathe Subtil

**Affiliations:** ^1^Unité de Biologie Cellulaire de l'Infection Microbienne, Institut PasteurParis, France; ^2^Centre National de la Recherche Scientifique UMR3691Paris, France; ^3^Unité de Biochimie des Interactions Macromoléculaires, Institut PasteurParis, France; ^4^Centre National de la Recherche Scientifique ERL6002Paris, France

**Keywords:** reporter, secretion signal, exoproteome, secretion machinery, live imaging

## Abstract

Bacteria have acquired multiple systems to expose proteins on their surface, release them in the extracellular environment or even inject them into a neighboring cell. Protein secretion has a high adaptive value and secreted proteins are implicated in many functions, which are often essential for bacterial fitness. Several secreted proteins or secretion machineries have been extensively studied as potential drug targets. It is therefore important to identify the secretion substrates, to understand how they are specifically recognized by the secretion machineries, and how transport through these machineries occurs. The purpose of this review is to provide an overview of the biochemical, genetic and imaging tools that have been developed to evaluate protein secretion in a qualitative or quantitative manner. After a brief overview of the different tools available, we will illustrate their advantages and limitations through a discussion of some of the current open questions related to protein secretion. We will start with the question of the identification of secreted proteins, which for many bacteria remains a critical initial step toward a better understanding of their interactions with the environment. We will then illustrate our toolbox by reporting how these tools have been applied to better understand how substrates are recognized by their cognate machinery, and how secretion proceeds. Finally, we will highlight recent approaches that aim at investigating secretion in real time, and in complex environments such as a tissue or an organism.

Secretion refers to the capacity, shared by all cells, to release a selected subset of the proteins they produce beyond the membrane that defines them as individual entities. In bacteria, secreted proteins are implicated in many essential functions such as nutrient uptake and catabolism, biodegradation of polymers, respiration, motility, cell attachment to the substratum or to other cells to allow beneficial or detrimental contacts, and biofilm formation. In pathogenic bacteria, the major virulence factors are typically secreted into the milieu or injected into neighboring target or host cells to change their integrity or function. These multiple roles are often essential for bacterial fitness, and several secreted proteins have been studied as potential drug targets. Optimization of the secretion process is also key for the production of many bioengineered products. Protein secretion, which allows communication with the external world, is also a fascinating biological problem for which numerous mechanisms have evolved, each one adapted to different, and often multiple, biological functions.

The purpose of this review is to provide an overview of the available biochemical, genetic and imaging tools that have been developed to evaluate protein secretion in a qualitative or quantitative manner. The different secretion machineries in bacteria are briefly presented in Figure 1 and we refer the reader to several excellent reviews for mechanical insight on these processes (Desvaux et al., 2009; Korotkov et al., 2012; Leyton et al., 2012; Lycklama A Nijeholt and Driessen, 2012; Palmer and Berks, 2012; Kanonenberg et al., 2013; Christie et al., 2014; Ho et al., 2014; Basler, 2015; Costa et al., 2015; Ates et al., 2016; Green and Mecsas, 2016). Importantly, different tools are applicable to different secretion systems, and the choice of tools is oriented by the question asked. Therefore, after a brief overview of the different tools available, we will illustrate their advantages and limitations through a discussion of some of the current open questions related to protein secretion. We will start with the question of the identification of secreted proteins, which for many bacteria remains a critical initial step toward a better understanding of their interactions with the environment. We will then illustrate our toolbox by describing how these tools have been applied to better understand how substrates are recognized by their cognate machinery, and how secretion proceeds. Finally, we will highlight recent technical developments that aim at investigating secretion in real time and in complex environments such as a tissue or an organism. For space limitations, we will not discuss here the tools to study pilus assembly systems, wherein secretion is coupled to polymerization of protein subunits into fibers, and which include type I pili (Remaut et al., 2008), type IV pili (Berry and Pelicic, 2015), or curli (Van Gerven et al., 2015).

**Figure 1:**
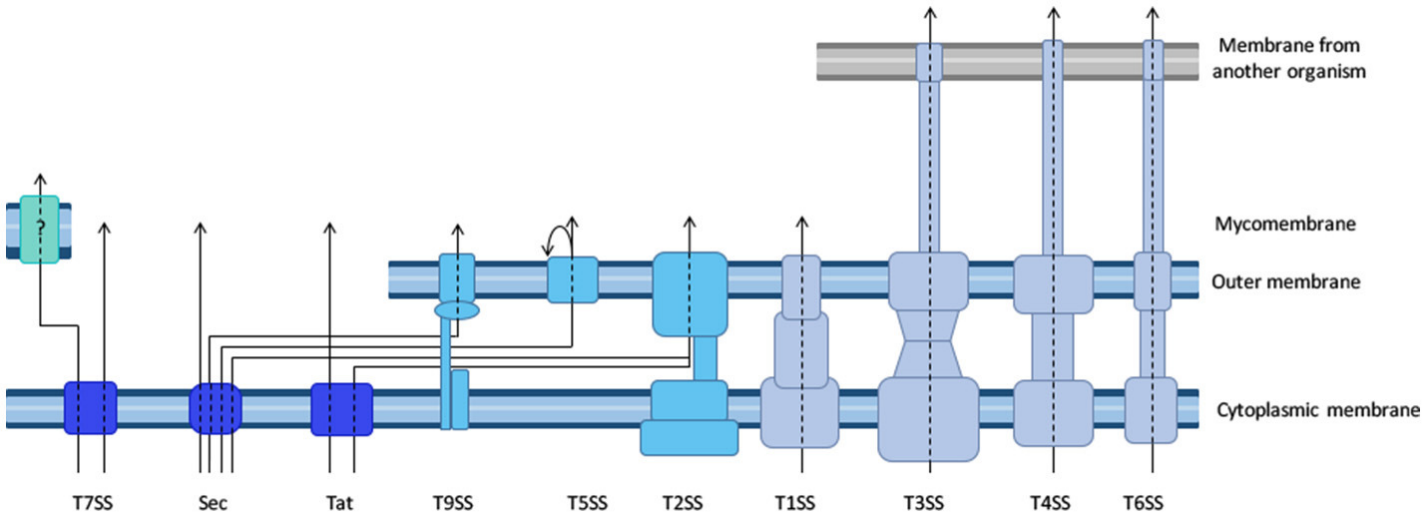
Diversity of the bacterial secretion systems. Schematic representation of the secretion systems identified in bacteria, partially based on structural data. In monoderm bacteria **(left)**, protein export (synonymous for secretion in that case) follows the Sec or Tat pathway, or the signal peptide independent T7SS. In diderm-non-LPS bacteria such as *Mycobacteria* or *Corynebacteria* **(far-left)**, it is unknown whether the T7SS system results in protein secretion in one or two steps (question mark). In diderm-LPS bacteria **(center and right)**, secreted proteins can reach the external environment through a one-step process via T1SS, T3SS, T4SS, or T6SS. Other secreted proteins are first exported to the periplasm via the Sec system (T2SS, T5SS, or T9SS) or the Tat system (for T2SS only).

To discuss protein transport across bacterial membranes, we need to clarify the terminology, as the term “protein secretion” is commonly used to describe three distinct processes. We will use the term “export” to describe the translocation of proteins across the cytoplasmic membrane (also called inner membrane (IM) in diderm bacteria). In monoderm bacteria, exported proteins are surface exposed, thus export is equivalent to “secretion.” However, in diderm bacteria, exported proteins typically remain intracellular, and are therefore not “secreted,” unless another machinery takes them across the outer membrane (OM). Several trans-envelope machineries (classified by numbers, often reflecting the order of their discovery) can perform this second translocation step, including the Type 2, 5, 7, and 9 secretion systems (T2SS, T5SS, T7SS, and T9SS). Other secretion machineries that span the entire envelope of diderm bacteria promote protein secretion in a single step directly from the cytoplasm (T1SS, T3SS, T4SS, and T6SS). Note that some secreted proteins may remain associated with cell surface, and are therefore not synonymous with the “exoproteome” that describes the subset of proteins present in the extracellular medium (Desvaux et al., 2009). Finally, the third process commonly covered by the term “secretion” leads to the injection of the protein beyond a third membrane, that of a neighboring cell, so that the translocated protein becomes inserted in this membrane or is released in the cytoplasm of the neighboring cell. While this process is more accurately described by the word “injection,” the term secretion is also suitable.

## Overview of the toolbox

The different methods to investigate protein secretion follow two main strategies. In the first, secreted proteins are identified after a fractionation step that isolates the compartment into which they are targeted. The second strategy is to keep cells intact, and use assays based on accessibility to probes, or on the activity of the secreted proteins, to monitor secretion. This second category includes several microscopy-based assays, and is amenable to the study of secretion in living cells. It also includes genetic or chemical screens aimed at the identification of components of secretion machineries and of secretion signals (see Identification of components of secretion machineries and secretion signals). Complementary to these approaches, bioinformatics tools are being used to identify putative secretion substrates based on their sequence (see Bioinformatics tools).

### Fractionation-based assays

Fractionation-based assays are most appropriate to study proteins that are released from the bacteria, either free in the extracellular medium, or injected into a host cell. However, coupled to strategies to purify the OM, such assays can also be used to study proteins that remain associated with the bacteria after secretion.

The fractionation step can be very straightforward, like separating bacteria from the culture medium by centrifugation, or more complicated, for instance in the case of a protein translocated into a given eukaryotic compartment, that will require isolation of that compartment. The readout for secretion after the fractionation step depends on the protein of interest (detection with antibodies, detection of localized enzymatic activity, use of an engineered chimera with a readily-detectable reporter protein etc.), if the strategy is used to follow a given protein. Progress in protein identification by mass spectrometry now enables the use of fractionation-based strategies to characterize bacterial exoproteomes and to detect protein modifications after secretion, without prior information on the secretion mechanism involved. One limitation of this strategy is that setting up the appropriate protocol for the isolation of the compartment of interest to limit contamination by proteins from other compartments can be time-consuming. The high sensitivity of the technique raises the issue of false-positives and requires further experimental validation. For example, a bacterial protein can only be considered as secreted if most of it is found in an extra-cytoplasmic compartment or environment and if such a location is compatible with its function. Furthermore, identification of membrane proteins by mass spectrometry remains technically challenging, and the approach is therefore best suited for soluble secreted proteins.

### Whole-cell based assays

Identification and characterization of secretion system components usually starts with genetic analyses such as deletion or insertion mutagenesis that can define the roles of individual components in more or less complex secretion machineries. Ideally, secretion should be specifically linked to a phenotype to allow for screening or selection; for example, secretion of an amylase is required for growth on starch, while secretion of a hemolysin produces a halo on blood agar plates (see Analysis of protein secretion signals and machineries for examples of genetic and chemical screens). Other, more complex phenotypes can be studied *in vitro*, such as killing of target bacteria *via* the T6SS effectors in mixed bacterial cultures (Brunet et al., 2013) (see When is protein injection activated?). Appearance of a given protein on the bacterial surface can sometimes be assessed directly using antibodies or reporter systems, as illustrated below. For proteins secreted into the extracellular medium, or translocated into a neighboring cell, use of a reporter system is usually the method of choice, in particular when secretion is measured using microscopy to achieve spatial and temporal resolution.

## Identification of secreted proteins

Secreted proteins are ambassadors, mediating most of the interactions of a bacterium with its surrounding environment. Cataloguing the secreted proteins is often an obligatory step toward a comprehensive understanding of how a given bacterium deals with its environment. Some of the tools that can be used to identify secreted proteins, like the bioinformatics approaches described below, are specific to a given secretion machinery. Others, like proteomics-based approaches, or phage display, do not require information on the secretion mechanism. The tools illustrated below are complementary. Typically, global approaches generate lists of secreted proteins candidates, which are later validated using targeted secretion assays, often based on reporter fusion systems.

### Bioinformatics tools

Type 1 to type 6 secretion systems (Figure 1) are sufficiently well documented and conserved to predict the secretion machinery repertoire in newly sequenced bacterial genomes. One recent study built online and standalone computational tools to predict protein secretion systems and related appendages accurately in bacteria with an OM containing lipopolysaccharide, retrieving ~10,000 candidate systems amongst which T1SS and T5SS were by far the most abundant and widespread (Abby et al., 2016). The identification of the substrates of these secretion machineries is more difficult, and novel secretion substrates generally cannot be identified unambiguously from genomic sequence alone. However, in many cases, sequence similarity with a known secretion substrate, and/or the presence of a “signal peptide” (see below), and/or genomic localization in proximity to genes coding for a secretion machinery, provide strong indications of novel secretion substrates. This is often not sufficient, especially for secretion substrates of pathogenic bacteria that are tailored for a very specific target, and are therefore often specific to a single bacterial species. To identify these elusive secretion substrates, machine-learning approaches have been implemented for use with T3SS and T4SS, for which the data base is sufficiently large. Globally, secretion substrates fall into two categories, depending on the presence or absence of a so-called signal peptide.

#### First scenario: presence of a signal peptide

Two machineries export proteins across the IM: the Sec translocon and the twin-arginine translocation (Tat) machinery. Proteins that are targeted to these export machineries have N-terminal extensions called signal peptides. Canonical signal peptides have a tripartite structure with a basic region at the N-terminus, a central hydrophobic region and a polar carboxyl terminus with a consensus cleavage site (AXA) (von Heijne, 1990). The Tat signal peptides differ somewhat from the Sec- targeting signals in that they possess an extended N-terminal region with a conserved twin-arginine motif TRRxFLK that is crucial for targeting to Tat export pathway (Palmer and Berks, 2012). Importantly, the Tat pathway is capable of transporting folded proteins and protein complexes; therefore, proteins that lack a signal peptide but form complexes with partner subunits that have twin-arginine signal peptides can also be exported in a “piggy-back fashion” through this pathway. Furthermore, it is important to note that some bacterial genomes have a strong base compositional bias and, consequently, encode Sec-dependent proteins with non-canonical signal peptides (Payne et al., 2012).

Several bioinformatics programs can be used to predict the presence of cleavable Sec or Tat signal peptides, such as SignalP (http://www.cbs.dtu.dk/services/SignalP) (Petersen et al., 2011), PSort (http://www.psort.org), which conveniently also provides a list of links toward other subcellular prediction programs, Pred-Tat, TatP or TatFind (see Berks, 2015, for comparison). Lipoprotein signal peptides are a distinct class of Sec dependent signal peptides characterized by a C-terminal consensus sequence, the lipobox, which ends with an absolutely conserved cysteine residue that, after fatty acylation, becomes the first residue of the mature protein (www.cbs.dtu.dk/services/LipoP/) (Juncker et al., 2003). Experimental validation is necessary, however, to demonstrate a tentatively-identified secreted protein uses a given transport pathway to exit the producing cell, as illustrated in parts Analysis of protein secretion signals and machineries and Resolution of secretion in time and space.

As explained earlier, protein transport across the IM by the Sec or Tat pathways in monoderm bacteria results in protein secretion. However, in diderm bacteria, proteins face an additional barrier, the OM that, together with the IM, defines the periplasmic compartment with distinct properties, composition and content. While the periplasm is the final destination for many proteins, others are inserted into the OM or cross it entirely to reach the bacterial surface using specialized transport systems. Protein secretion pathways that include a periplasmic intermediate are called two-step pathways.

The T2SS is a typical two-step pathway that takes up specific periplasmic substrates in a folded state. T2SS substrates can therefore be recognized by the presence of an N-terminal signal sequence in their precursors. However, despite many extensive analyses, the recognition events and signals that mediate the second transport step have not been elucidated and appear to vary between different bacterial species and substrates (Korotkov et al., 2012).

T5SS substrates, formerly called auto-transporters, are made of a translocator and passenger domain; these domains are usually encoded by a single gene, but can also be separate polypeptides in the so-called two-partner secretion systems (Leyton et al., 2012). The conserved and mandatory C-proximal translocator domain with characteristics typical of most outer beta-barrel membrane proteins, following a large N-proximal domain with a signal sequence is usually sufficient to predict the latter as the passenger (secreted) domain (Abby et al., 2016).

Another two-step secretion pathway is the recently discovered T9SS, found exclusively in the Bacteroidetes phylum. Substrates of this pathway are secreted in a folded state, and, in addition to the signal sequence, share a conserved C-terminal domain harboring the secretion signal (Sato et al., 2010; de Diego et al., 2016).

#### Second scenario: absence of a signal peptide

Proteins that are not made as precursors with an amino terminal signal peptide can still be secreted, by T1SS, T3SS, T4SS, T6SS, or T7SS. It proved difficult or impossible to identify sequence features in secretion substrates that indicate that they will use one or other of these pathways. Several machine-learning techniques have been developed recently for this purpose based on datasets of known T3SS and T4SS secretion substrates. They differ in terms of the machine learning methods, the curated data sets and the features used, and reach different levels of prediction performance. Several, but not all (Meyer et al., 2013), of these tools were recently compared (An et al., 2016). All predictors are flawed, to various degrees, with false positives (secretion signal identified in non-secreted proteins) and false negatives (documented secretion substrates not predicted as such), so while they can be precious in orienting research, experimental validation is required.

T1SS substrates also contain a Sec-independent secretion sequence, which is either located at the N-terminus (certain bacteriocins or colicins) or at the C-terminus (all other systems) of the substrate. Like in T3SS and T4SS substrates, these sequences lack recognizable features, so T1SS substrates cannot be identified based on the recognition of a characteristic secretion signal (Kanonenberg et al., 2013). However, prediction tools cannot yet be developed, because the data base is too small to build training sets. The same is true for T6SS substrates for which some properties (size, isoelectric point, operon structure) have been used to orient genome searches, but robust bioinformatics based methods do not exist (Ho et al., 2014).

Finally, the T7SS, initially identified in mycobacteria, is still poorly understood. Mycobacteria and related genera have an external membrane composed of unique and complex lipids and are therefore diderm, despite the fact that, like monoderms, they stain Gram-positive. They secrete several proteins by a Sec-independent mechanism, and it is not known whether protein secretion occurs in one or two steps. Substrates of this secretion machinery share a loosely defined C-terminal secretion signal, which includes the consensus motif YxxxD/E (Ates et al., 2016). The T7SS is also present in a few monoderm bacteria such as *Staphylococcus aureus*, where it promotes secretion of proteins with antibacterial activities (Cao et al., 2016).

### Identification of secreted proteins through proteomics

One method of choice to identify new secreted proteins in the extracellular medium, irrespective of the secretion mechanism, is mass spectrometry. Combined with the use of mutant strains, or specific culture conditions, it can also identify the substrates of a given secretion machinery. For instance, comparison of the exoproteome of *Pseudomonas aeruginosa* in conditions where one T6SS machinery was on or off allowed the identification of three novel T6SS secretion substrates (Hood et al., 2010).

Quantitative proteomics not only can lead to identification of novel effectors, but also provide information on the regulation of secretion, as was recently shown with a focused exoproteome analysis of the T3SS in *Ralstonia*. The use of secretion mutants revealed that secretion is finely tuned and identified specific subsets of effectors with different secretion patterns (Lonjon et al., 2016).

Combined with a fractionation method to isolate a specific compartment of a target cell, proteomics can also identify novel translocated proteins in their target location. For example, this approach was used to draw up a list of putative nuclear effectors of *Anaplasma phagocytophilum* (Sinclair et al., 2015), and to identify *Chlamydia trachomatis* proteins associated to lipid droplets (Saka et al., 2015). Obviously, the method is not sufficiently sensitive if the effector is present in minute amounts and is overwhelmed by eukaryotic proteins. One way to circumvent this limitation is to enrich for bacterial proteins using non-canonical amino acid tagging (Mahdavi et al., 2014). Selective labeling of bacterial proteins is accomplished via translational incorporation of a methionine surrogate. The technique requires the introduction of a gene coding for a mutant form of the methionyl-tRNA synthetase into the bacterium. During mixed culture with the bacterium, the host cells do not produce the altered methionyl-tRNA synthetase and do not incorporate the methionine surrogate, whereas the bacteria do. Bacterial proteins with the methionine surrogate are then enriched from the eukaryotic cells and identified by mass-spectrometry. This approach was used to identify *Yersinia* proteins that were secreted into the medium and translocated into cells (Mahdavi et al., 2014). In addition, pulse labeling with the methionine surrogate can be used to achieve temporal resolution (see Resolution of secretion in time and space).

Integral membrane proteins are notoriously difficult to detect by mass spectrometry because detergents interfere with the analyses. In a pioneering work in which alkaline sodium carbonate was used instead of detergent, Molloy et al identified the majority of integral *Escherichia coli* OM proteins and several diacyl-glyceride attached lipoproteins (Molloy et al., 2000). Proteomics is currently the method of choice to obtain an overall view of the secretion capacity of a given bacterium. For instance, it was recently applied to characterize the OM proteome and the exoproteome of *Bacteroides fragilis*, highlighting striking differences with *Proteobacteria*, from which most of the current information on bacterial secretion was derived so far (Wilson et al., 2015).

### Reporter-based assays

A large part of the “secretion toolbox” relies on reporter-based assays, which use genetic tools to tag a given secretion substrate with a readily detectable but otherwise neutral reporter and follow its secretion through a dedicated assay. In most cases, these assays are applied with a specific secretion machinery in mind, which orients the choice of the tag and the tagging strategy. A few rules are listed below, and most tags currently in use are briefly described in Table 1, with examples of their application.

**Table 1:** Reporter systems to track protein secretion.

**Tag**	**Read-out**	**Tested for**	**Examples of application**
Calmodulin-dependent adenylate cyclase (Cya)	cAMP production,	T3S, T4S	Defining components and signals required for secretion (Sory and Cornelis, 1994)
	Western-blot		Genome wide screens for T3S and T4S effectors candidates (Subtil et al., 2005; Carey et al., 2011)
Alkaline phosphatase (PhoA)	Enzymatic assay		Protein export reporter (Manoil and Beckwith, 1985).
Amino-peptidase (AP)	Enzymatic assay	Sec dependent export	Optimization of secretion (Guan et al., 2016)
		(Gram+ bacteria)	
β-1,4-mannanase (ManB)	Enzymatic assay	Sec dependent export	Optimization of secretion (Lin et al., 2015)
		(Gram+ bacteria)	
staphylococcal nuclease (NucA)	Enzymatic assay	Sec dependent export	Validation of predicted signal peptides and optimization of secretion (Mathiesen et al., 2009)
		(Gram+ bacteria)	
Green fluorescent protein (GFP)	fluorescence	T2S	Assembly pathway of the T2S complex (Lybarger et al., 2009)
Split GFP	fluorescence	T3S	Localization of secreted effectors in eukaryotic host (Van Engelenburg and Palmer, 2010)
Tetracysteine motif	fluorescence	T3S	Kinetics of effector translocation (Enninga et al., 2005)
LOV	fluorescence	T3S	Detection of effector translocation (Gawthorne et al., 2016)
Glutamyl carboxypeptidase	fluorescence	T3S	Detection of protein secretion (Yount et al., 2010)
TEM-1 β-lactamase	fluorescence (FRET)	T3S, T4S	Genome wide screen for T4S effector candidates (Zhu et al., 2011), study of translocation dynamics (Mills et al., 2013)
Gaussia princeps luciferase (Gluc)	luminescence	T1S	Detection of secreted fusion protein in culture supernatant (Wille et al., 2012)
Bacteriophage P1 Cre recombinase	luminescence/fluorescence	T3S, T4S	Detection of effector translocation (Luo and Isberg, 2004; Briones et al., 2006)
Phosphorylation target	Western blot: detection of phosphorylated tag	T3S, T4S	Detection of effector translocation (Day et al., 2003; Garcia et al., 2006)
Nucleoskeletal-like protein (Nsp)	Western-blot	Flagellar secretion apparatus	Identification of export signal (Wang et al., 2016)

#### Tag flexibility

**Figure 2:**
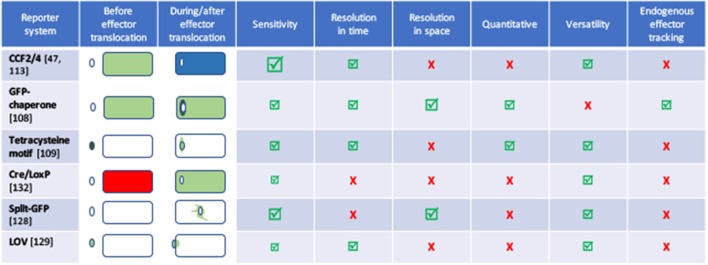
Comparison of the different tools to image effector secretion into living cells. Illustrations reflect different experimental set-ups, please refer to the indicated reference for description of the bacterium, effector and time scale. Bacteria are represented by an oval, cells by a rectangle. Colors represent the fluorescent signal recorded before **(left)** and after **(right)** the effector translocation has started. Discussion on the pros and cons of some of these assays can be found in Ehsani et al. (2009) and Zuverink and Barbieri (2015).

For each new tag, one prerequisite is to ensure that the tag itself is neutral with regard to the secretion capacity of the machinery under inspection. Some secretion processes require the secretion substrate to unfold, and proteins that fold very rapidly upon synthesis are inappropriate as tags. For instance, green fluorescence protein (GFP) (Jaumouille et al., 2008) and glutathione S-transferase (Riordan et al., 2008), which fold rapidly block T3SS. Although GFP-tagged proteins are successfully exported through the Sec pathway, GFP fluorophore is inefficiently folded in the periplasm (Feilmeier et al., 2000). On the other hand, GFP is an ideal reporter for the Tat system, since it can be exported in a folded form (Thomas et al., 2001). The GFP re-folding problems have been solved using superfolder GFP variant (Choi et al., 2017) or monomeric red fluorescent protein (mRFP) and its derivatives (Shaner et al., 2004). Several strategies were developed to find alternatives to GFP to facilitate the tracking of effector injection into a host cell with fluorescence (Figure 2, and see part Resolution of secretion in time and space for live imaging with temporal resolution).

#### Tag position

The position at which the tag is fused to the secreted protein is important because the tag can compromise the recognition of the secretion signal. For instance, T3SS substrates have an amino-terminal signal that must remain unaltered in the tagged construct. Typically, C-terminal tags are used for proteins with an N-terminal signal sequence.

#### Secretion readout

Many tags are associated with an enzymatic activity, which provides quantitative data on secretion. Tags that are enzymatically active only in a given environment facilitate quantification. For example, alkaline phosphatase and beta-lactamase are active only in the periplasm, and have been used extensively to probe IM protein topology and to identify periplasmic proteins by gene fusion approaches. The membrane impermeable beta-lactamase substrate nitrocephin is a useful probe for surface exposed beta-lactamase fusions that remain cell associated upon secretion (Sauvonnet and Pugsley, 1996). One widely used reporter of protein injection (via T3SS or T4SS) into a host cell is the calmodulin-dependent adenylyl cyclase of *Bordetella pertussis*. Since bacteria do not produce calmodulin, whereas eukaryotic cells do, accumulation of cyclic AMP marks the injection of the reporter in the cytoplasmic compartment of the target cell (Sory and Cornelis, 1994). Reporter tags that become phosphorylated in the eukaryotic cytosol have also been used (Day et al., 2003; Garcia et al., 2006). In the last ten years, the development of fluorogenic beta lactamase substrates allowed to follow injection of effectors fused with beta lactamase (TEM1), and this enzymatic-based assay has also largely been used, including very recently, *in vivo* (see Resolution of secretion in time and space).

In contrast to global approaches using bioinformatics or proteomics described above, reporter-based assays are only amenable in genetically tractable microorganisms. However, secretion machineries and secretion signals are well conserved, allowing the use of heterologous secretion systems to identify secretion substrates in non-genetically tractable bacteria (Subtil et al., 2001).

Reporter-based assays are mostly used for candidate-based approaches, because they require the generation of genetically modified organisms. They are typically used to validate candidates indicated by secretion signal predictors or by proteomic approaches. However, high-throughput cloning strategies and simplification of the read-outs have allowed the application of these assays to screen for novel secretion substrates in genome wide approaches (Subtil et al., 2005; Carey et al., 2011; Zhu et al., 2011).

### Functional screens in yeast

Many proteins injected into a eukaryotic host cell target proteins and pathways that are highly conserved in all eukaryotic cells. Based on this observation, expression libraries of bacterial genes under inducible promoters have been screened in yeast. Most screens selected bacterial genes that inhibited yeast growth (Campodonico et al., 2005; Slagowski et al., 2008), but atypical localization of bacterial proteins (for instance in the nucleus) (Sisko et al., 2006) or other particular phenotypes such as interference with the secretory pathway can also be screened for (Heidtman et al., 2009). The ease of manipulating yeast genome, and conservation of the molecular pathways with higher eukaryotes, can then facilitate the identification of specific targets of the bacterial effectors identified by this approach.

### Phage display technology

Jacobsson and Frykberg were the first to take advantage of the power of shot-gun filamentous bacteriophage display (display of random fragments of bacterial genomic DNA) to identify *S. aureus* proteins that interact with components of the extracellular matrix and immunoglobulins (Jacobsson and Frykberg, 1996). Since then, many genes encoding proteins involved in host-microbial interactions have been identified using this technology. Coupled to next generation sequencing, it can also be applied to the identification of secretomes, in particular in mixed microbial populations. This powerful technology was recently reviewed in detail and will not be discuss further (Gagic et al., 2016).

## Analysis of protein secretion signals and machineries

### Identification of components of secretion machineries and secretion signals

#### Genetic and chemical screens

**Figure 3:**
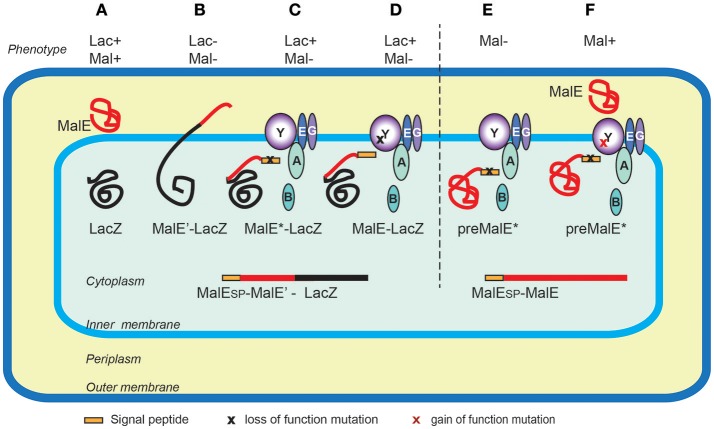
Genetic selections of export-defective mutants based of MalE-LacZ fusions. In wild type *E. coli* the correct localization of LacZ (cytoplasmic) and MalE (periplasmic) allows lactose catabolism and maltose uptake, respectively, conferring the ability to ferment these sugars, and the red colony phenotype on MacConkey indicator plates **(A)**. The MalE-LacZ fusion proteins directed to the periplasm confer a Lac- phenotype **(B)**, which served as a basis for selection of spontaneous Lac+ mutants on lactose tetrazolium plates. These strains either contained mutations in MalE signal sequence (MalE^*^) **(C)**, or the loss-of- function mutations in the *sec* genes encoding export factors, five of which, (SecA, B, Y, E and G) are depicted **(D)**. Bacteria producing the full-length MalE precursor with a signal sequence mutation are export defective and Mal- **(E)**, allowing for selection of Mal+ suppressor mutations (gain-of-function *prl* alleles) mapping in several *sec* genes, (e.g., *secY*) that promote export of MalE^*^ variants with signal sequence mutations **(F)**. Note that the MalE signal sequence (yellow rectangle) is absent from periplasmic MalE-LacZ or MalE, as it is cleaved and degraded upon export across the IM.

The first protein export signals identified were the cleavable N-terminal signal peptides that target proteins to the endoplasmic reticulum in eukaryotic cells (Jackson and Blobel, 1977). In *E. coli*, studies of genes involved in maltose and lactose transport or catabolism have provided a wealth of genetic tools to study protein export (Figure 3). Powerful genetic approaches have been designed based on special properties of beta-galactosidase (LacZ) fusions (Shuman and Silhavy, 2003). Selections devised to identify mutations that affect protein export were based on the Lac- phenotype of strains producing fusions of the periplasmic maltose-binding protein MalE with the cytoplasmic LacZ. These fusions, which target beta-galactosidase to the export pathway, are enzymatically inactive and confer a Lac- phenotype to bacteria, allowing for selection of Lac+ export-defective mutants. The mutations frequently mapped to the proximal region of gene fusions coding for the signal peptide (Bedouelle et al., 1980), introducing charged residues in their hydrophobic segment. Importantly, Lac+ selection also yielded several classes of mutants with pleiotropic export defects. Many of those were conditional lethal mutations in genes encoding novel protein export factors, including the preprotein translocase SecA, or the translocation channel SecY (Ito et al., 1983). Suppressor (*prl*) mutations that restored export of proteins with defective signal sequences also mapped in the *secA* and *secY* genes, strongly suggesting that signal sequences interacted with their gene products. Indeed, this was confirmed by many studies and by structural data that revealed how signal sequences bind to SecA (Gelis et al., 2007) or SecY and the lipid bilayer (Li et al., 2016). Although they do not share sequence homology and appear to be interchangeable between proteins, signal sequences differ in their ability to promote efficient export, as illustrated in a genome-wide study of *Lactobacillus* signal peptides that assessed their ability to promote export of nuclease reporter (Mathiesen et al., 2009). Successful approaches to improve signal sequence efficiency have been reported. For example, combinatorial mutagenesis of the signal sequence-coding region resulted in variants with increased export and production of beta-lactamase, probably due to an overlap of export signals with elements of translational or post-translational regulation (Heggeset et al., 2013).

The Tat signal was also largely investigated through genetic screens. Although a native Sec substrate, MalE was successfully used to monitor the activity of the Tat-dependent signal peptide of TorA, by following maltose utilization on pH indicator media (indicating maltose fermentation) or by growth on minimal maltose plates (Kreutzenbeck et al., 2007). This allowed the use of powerful genetic approaches to identify suppressors of Tat signal sequence changes that restored MalE export, affecting genes encoding the TatB and TatC export machinery components (Lausberg et al., 2012). In a genome-wide screen for Tat-dependent exo-proteins of *P. aeruginosa*, the authors used the *E. coli* amidase AmiA as a reporter to validate the functionality of a newly identified Tat signal peptide (Ball et al., 2016). Tat-mediated AmiA export is required for the correct separation of daughter cells during cell division, and defects in this process render *E. coli* hypersensitive to detergents, providing a simple plate test.

In monoderm bacteria, surface proteins of the “LPxTG” family are anchored to the cell wall in a process mediated by the sortase enzymes (Schneewind and Missiakas, 2014). Since sortase substrates include major virulence factors, small molecule screening has been used to identify sortase inhibitors (Maresso et al., 2007). In *S. aureus*, transposon mutagenesis was used to look for mutants with defective surface anchoring of the protein A (SpA), using detection of fluorescently labeled Anti-SpA antibodies and flow cytometry (Frankel et al., 2010).

#### Assays of protein export to the periplasm or secretion across the OM

##### Signal pepides and the Sec system

**Figure 4:**
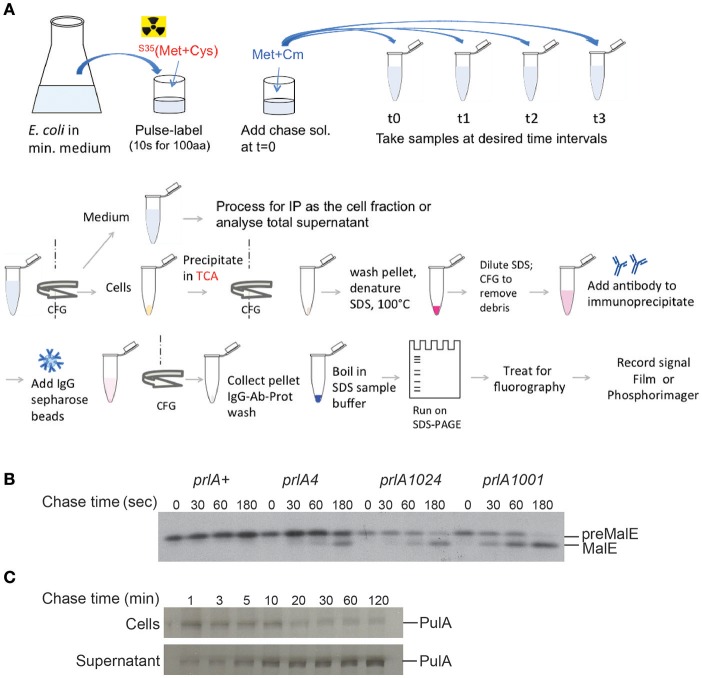
Pulse-chase assay to analyze protein export or secretion rates. **(A)** Bacteria are grown in minimal medium in conditions inducing the expression of the gene of interest and pulse labeled with ^35^S-methionine and cysteine for a short period (30 s–2 min, depending on the size of the protein under study), followed by addition of cold methionine and chloramphenicol (Cm) to stop protein synthesis. Samples are collected at indicated times and bacterial cultures are either precipitated with TCA or separated from the medium prior to precipitation of each fraction. The collected precipitates are washed with acetone, dissolved in SDS buffer and boiled to denature proteins. Upon the removal of cell debris by centrifugation and dilution of SDS, antibodies are added for immuno-precipitation. Antigen-antibody complexes are adsorbed on protein A-sepharose beads, washed and eluted in SDS sample buffer for analysis by SDS-gel electrophoresis and fluorography. **(B)** Kinetics of signal sequence processing in preMalE variant carrying a signal sequence mutation, reflecting the kinetics of MalE export. While the preMalE export and processing are blocked in wild type *E. coli* (*prlA*+), they are partially restored in strains carrying different suppressor *prlA* alleles of the *secY* gene encoding the translocation channel. Bacteria were labeled for 20 s with radioactive methionine and a chase with excess cold methionine was performed for the indicated times. After immuno-precipitation of total cell extracts with anti-MalE antibodies, proteins were separated on SDS-PAGE, and analyzed by fluorography (modified from Francetic et al., 1993). **(C)** Pulse-chase and fractionation were used to follow the kinetics of pullulanase (PulA) secretion *via* the T2SS. The bacteria were cultured as in **(A)** and pulse-labeled for 3 min. Samples were collected after the indicated times of chase with cold methionine, and cell and supernatant fractions were separated by centrifugation, prior to immunoprecipitation and SDS-PAGE analysis as in **(A)** (modified from Francetic and Pugsley, 2005).

Measuring the rate of signal peptide processing can serve as a good quantitative indicator of protein export, as the signal peptidase cleavage takes place in the periplasm. Radioactively labeled amino acids (usually ^35^S - labeled methionine and cysteine) are incorporated into the newly synthesized proteins during a short “pulse” labeling period. Addition of an excess of unlabeled amino acids and an inhibitor of protein synthesis during a “chase” period allows one to follow precursor cleavage with precision and to establish the time course characteristic for a given strain (Figure 4; Kumamoto and Gannon, 1988). In addition, strong export defects, such as those caused by signal sequence mutations, can be observed in steady state by the presence of precursor forms of a protein by denaturing SDS-PAGE and immuno-detection. Fractionation in steady state can reveal partitioning of the precursors with the cell fraction and mature forms to the periplasm (Francetic et al., 2007). This approach has been used to characterize the strongly hydrophobic signal sequences that direct proteins like thioredoxin or DsbA to the co-translational export *via* the signal recognition particle (SRP) (Huber et al., 2005). All of these assays are target-based and rely on the availability of specific antibodies or antigen tags for immuno-precipitation of radioactively labeled protein or for immuno-detection.

##### Lipoproteins

Lipoproteins are exported proteins that undergo fatty acylation during biogenesis and remain anchored in membranes (Zückert, 2014). Lipoprotein signal peptides can be predicted by bioinformatics approaches, mostly thanks to the presence of a conserved lipobox motif with a consensus LAGC. This motif probably interacts with Lgt (Pallier et al., 2012), the first component of the biogenesis pathway that adds the diacyl-glycerol moiety to the invariant Cys residue. In the next step, the signal peptide is removed by a dedicated lipoprotein signal peptidase, Lsp. Accumulation of unprocessed precursors in the presence of globomycin, an Lsp inhibitor, can serve as a tool for experimental validation of predicted lipoproteins, at least in *E. coli*. Another method frequently used to demonstrate protein fatty-acylation *in vivo* is metabolic labeling with radioactive fatty acids, usually palmitate (Jackowski and Rock, 1986).

In *E. coli* and related bacteria, lipoproteins either remain anchored in the IM or are taken to the OM *via* the Lol sorting machinery (Zückert, 2014). The sorting signals that determine retention in the IM or extraction and transport to the OM typically reside within the four N-terminal residues of the mature lipoprotein. Systematic analysis of model lipoproteins in an *in vitro* membrane release assay allowed Hara and co-workers to characterize these signals in *E. coli* providing the basis for bioinformatics predictions (Hara et al., 2003). However, the sorting rules are not valid for all bacteria and there are many exceptions, even in well-studied enterobacterial species. Membrane fractionation in sucrose density gradients followed by immuno-detection is a reliable tool to determine *in vivo* localization of lipoproteins, as for integral membrane proteins. This method relies on differences in composition and density of the *E. coli* IM and OM, allowing good separation using equilibrium centrifugation in flotation sucrose density gradients, where the total membranes are deposited on the bottom and float upon ultra-centrifugation to their equilibrium density. Relative positions of specific proteins in gradient fractions can be analyzed by immunodetection, as in the systematic analysis of +2 residue substitutions in a model lipoprotein lipoMalE (Seydel et al., 1999). In addition, lipoMalE conferred a Mal+ phenotype to *E. coli* when localized in the IM but not in the OM, providing a plate assay for lipoprotein localization based on utilization of maltose as a carbon source. As a different approach, *in vivo* analysis of lipoproteins fused to the monomeric fluorescent protein mCherry can be performed following plasmolysis, which swells the periplasm and facilitates direct visualization of IM or OM lipoprotein association: while OM lipoproteins follow the contours of the bacterial cell, the irregularly shaped plasmolysis bays are decorated with IM associated lipoproteins (Lewenza et al., 2006).

While lipoproteins represent an important group of surface proteins in monoderm bacteria, in diderms they generally reside in the periplasmic membrane leaflets. However, recent investigations have revealed lipoproteins that are fully or partially surface-exposed, either through known secretion systems (T2SS, T5SS) (Leyton et al., 2012; Rondelet and Condemine, 2013), or by novel mechanisms (Wilson and Bernstein, 2016). In the Lyme disease agent *Borrelia burgdorferi* many lipoproteins that play a role in virulence are exposed on the cell surface. Their localization has been studied using mRFP as a fluorescent reporter. Using the model lipoprotein OspA-mRFP, a cell-sorting based mutant screen led to identification of residues required for the surface exposure presumably participating in a flipping step whose cellular determinants remain to be identified (Kumru et al., 2010). Some *E. coli* lipoproteins, including Lpp (Cowles et al., 2011) and RcsF, are partially exposed on the bacterial surface. This unusual feature has been tested using the so-called epitope walking approach of the OM lipoprotein RcsF. The FLAG tag was inserted at multiple positions in the RcsF sequence, and a dot blot analysis of whole and lysed cells using monoclonal anti-FLAG antibody allowed for identification of protein regions that are accessible in intact cells. The FLAG appears to be a useful and neutral tag that does not seem to interfere with transport across membranes (Konovalova et al., 2014). This approach revealed how RcsF uses OM proteins as portals for surface exposure, and how this mechanism allows the bacteria to monitor the functional state of their Beta-barrel Assembly Machinery (BAM) (Konovalova et al., 2014).

##### Surface exposed and associated proteins

A subset of exported proteins is inserted in the outer membrane and partially exposed on the cell surface to perform diverse functions, including solute uptake, macromolecule transport or proteolysis. The OM insertion generally relies on the signals encoded in the mature protein, notably the propensity to form beta-barrels, and is generally mediated by the BAM complex (Voulhoux et al., 2003; Wu et al., 2005). The BAM machinery is probably responsible for OM insertion of the beta-barrel domain of T5SSs, which provide the portal for secretion of so-called “passenger domains” (Leyton et al., 2012; Bernstein, 2015). Although the passenger domains can be cleaved and released into the medium to perform diverse extracellular functions, many of them remain surface bound. The surface exposure of specific proteins or protein domains can be assessed through analysis of non-permeabilized cells by immunofluorescence or by analyzing protease accessibility in whole cells, with a comparative assessment of general protease susceptibility of the same substrate in lysed bacteria (Besingi and Clark, 2015). For enzymes that degrade biopolymers, plate assays for protease, lipase, cellulase or chitinase activities, to name a few, rely on the visualization of a halo zone of substrate degradation surrounding the secreting colonies. Secretion of one of the first identified T2SS substrates, the lipoprotein pullulanase (d'Enfert et al., 1987), has been assessed by growth on minimal media containing pullulan as the sole carbon source, through degradation of chromogen-tagged pullulan, or by semi-quantitative enzymatic assays that determine the fraction of hydrolytic activity present in intact compared to the lysed bacteria by measuring the reducing sugar as the reaction product. All these methods are semi-quantitative, end-point assays, which do not provide kinetic information on protein secretion.

Similar approaches have been used to study surface protein secretion via T5SS or the recently discovered T9SS. In *Porphyromonas gingivalis* the T9SS is required for the black pigment of colonies on blood agar, linked to heme acquisition mediated by secreted proteases gingipains. Substrates of this pathway are exported to the periplasm via the Sec pathway and their N-terminal signal peptide. Their secretion in a folded state requires a C-terminal signal within a specific beta-sandwich domain, which is able to promote secretion of folded GFP (de Diego et al., 2016).

### Mechanistic approaches

Understanding a secretion mechanism requires detailed structural knowledge of the secretion machinery, its composition, biogenesis and dynamics during interactions with the secreted substrate. A few assays have been designed to understand how secretion proceeds. They are complementary to microscopic observations of secretion machineries, itself an expanding field of research (Costa et al., 2015; Li et al., 2016). The question concerning energy requirements for the process has been addressed by depleting ATP or by dissipating the proton motive force, two main energy sources required for membrane transport (e.g., Possot et al., 1997). Many secretion systems use at least one ATPase as an essential component, and ATP hydrolysis as a mechanical force generator (Costa et al., 2015).

#### Capture of protein interactions and of intermediate states in the secretion process

In many systems, a bacterial two-hybrid approach is an excellent tool to map protein-protein interactions between dynamic components in secretion systems, in particular membrane embedded elements in trans-envelope complexes (Karimova et al., 1998). Fusions to cytoplasmic fragments of the CyaA reporter can effectively block some transport intermediates in the membrane and allow assessment of interactions that are transient in the native system. Examples include studies of complexes in T2SS (Nivaskumar et al., 2016) and T6SS (Logger et al., 2016).

Specific inhibitors of secretion can also be used to block secretion at a specific step (Moir et al., 2011). Chemical libraries have been used to identify compounds that specifically inhibit elastase and Plc secretion in *Pseudomonas* T2SS using a colorimetric enzymatic screen of culture supernatants (Moir et al., 2011). Once such inhibitors are identified, the major challenge is to identify their specific protein targets and modes of inhibition.

In T1SS and T3SS, substrates are secreted in an unfolded state and bulky domains fused to the substrate might block secretion, potentially providing important clues about intermediates in the secretion process. In T3SS, blocking the secretion using a GFP fusion with a secretion substrate revealed that secretion occurs at a cell pole (Jaumouille et al., 2008). A bulky “knot” region fused to different T3SS substrate allowed Dohlich and coworkers to demonstrate that the substrate passes through the T3SS channel (Dohlich et al., 2014).

In many cases substrates of blocked or incomplete secretion systems are degraded *in vivo* due to the absence of specific partners, chaperones or cellular structures. In T6SS, for example, the component of an inner tube HCP binds specific folded substrates in the bacterial cytoplasm and is required for their stability (Silverman et al., 2013). Folded substrate PulA of the T2SS (East et al., 2016) or TcpF secreted by the assembly system of TCP pili (Kirn et al., 2003) are also prone to degradation if their secretion is compromised. Since protein secretion efficiency is typically assessed in steady state by combining fractionation and substrate detection using antibodies or activity assays, it is important to keep in mind that proteolysis of non-secreted substrate may skew quantification of secretion efficiency. While the bulk of secretion-defective variants will be degraded, a small amount of extracellular protein that escapes degradation might give an impression of full secretion efficiency. The use of radiolabeling in pulse-chase assays coupled to fractionation might help overcome this problem (Figure 4C). Radioactive labeling is a powerful tool in secretion analysis due to its unsurpassed sensitivity. Selective labeling of bacterial proteins in cell culture has helped to identify proteins from enteropathogenic *E. coli* injected into eukaryotic cells *via* T3SS (Kenny and Finlay, 1995).

A number of gene fusion and mutagenesis approaches have been employed using different T2SS substrates to elucidate the molecular nature of the secretion signal and the component of the secretion machinery with which they interact. As already discussed, the sequence diversity of exoproteins, including those using the same secretion system, and systems makes this task difficult, however. In view of their heterogeneity, it is reasonable to assume that T2SS components that are interchangeable between systems do not make specific contacts with secretion substrates. A site-directed *in vivo* cross-linking approach has been used to identify secretion motifs in PelI and their interactions with the T2SS of the plant pathogen *Dickeya dadantii*. The unnatural photo-cross-linkable amino acid pBPA incorporated at specific sites in exoproteins was used as a tool to capture transient complexes *in vivo*, providing evidence for PelI binding to OutD forming the OM channel of the T2SS, and to OutC, which interacts with OutD (Pineau et al., 2014). A similar approach has been used to track secretion intermediates in T5SS (Ieva et al., 2011).

#### *In vitro* reconstitution

In an advanced stage of analysis, secretion systems could be reconstituted *in vitro* to gain insights into the transport process. With the notable exception of the Sec system that has been functionally reconstituted *in vitro* (Duong and Wickner, 1997), few other systems have been studied at this level, due to their complexity and difficulties to extract them from the bacterial envelope in a functional state. Nevertheless, *in vitro* transcription-translation systems have been used successfully to study biogenesis of specific transport components including the OM channel called secretin that self-assembles and insert into liposomes (Guilvout et al., 2008) or the IM prepilin peptidase, both components of the T2SS (Aly et al., 2013).

## Resolution of secretion in time and space

Several tools have been developed in the last two decades to improve the spatio-temporal resolution of techniques aimed at tracking secretion. They have mostly been applied to effector injection into a neighboring cell through the T3SS and T4SS, and to some extent to the T6SS.

### When is protein injection activated?

Protein injection in a neighboring cell is a highly regulated process that is typically constitutively turned off and only activated by specific signals. In many cases, activation occurs at least in part at the transcriptional level (Mavris et al., 2002; Urbanowski et al., 2005), so the activity of promoters has been used as a read-out for secretion activity. Monteiro et al. (2012) used the luciferase reporter under the control of the promoter of *hrpB*, the transcriptional regulator that controls the expression of *Ralstonia solanacearum* T3SS genes, in order to track the activity of the T3SS *in planta*. This approach revealed that T3SS activation was important not only for the first stages of infection, to manipulate host plant defenses, but also during late stages of infection (Monteiro et al., 2012). In a somewhat different set-up using a fast-maturing GFP under the control of the transcription activator MxiE, Campbell-Valois and collaborators provided evidence that *Shigella flexneri* T3SS goes through two waves of activation: one upon cell contact, during the invasion process, and a second, concomitant with the motile stage of the infection cycle, when bacteria move throughout the cytoplasm through actin cytoskeleton remodeling. The T3SS was switched off between these two phases (Campbell-Valois et al., 2014).

Another readout of active protein injection can be found in the effects on the target, when this effect is rapid and easy to detect. The T6SS functions as a contractile nanomachine, called the molecular crossbow, that punctures target cells to deliver lethal effectors. Time-lapse fluorescence microscopy of cocultures demonstrated that prey cells were killed upon contact with predator cells, and that prey lysis occurred within minutes after sheath contraction (Brunet et al., 2013). A killing assay was designed to understand how *Proteus mirabilis* coordinates multicellular swarming behavior and discriminates itself from another *Proteus* species during swarming. This assay, together with live-cell microscopy, demonstrated that T6SS-mediated lethality is unique to morphologically distinct swarmer cells, and that it requires direct cell-cell contact (Alteri et al., 2013).

The strategies illustrated above aim at measuring the activation of a given secretion machinery. They do not provide information on the nature of translocated effectors, or on the kinetics of secretion of a given effector, questions that are addressed below.

### How fast does protein injection take place and what is the hierarchy of substrate secretion?

Highly sensitive and time-resolutive tools were needed to answer these questions in order to focus on the very early secreted effectors, typically during the first 15 min following the attachment of a bacterium to its target cell. The current tools are mostly microscopy-based, allowing for single-cell measurements.

A pioneering work in the field focused on the secretion of SipA, an early effector of *Salmonella* Typhimurium (Schlumberger et al., 2005). The kinetics of secretion of this effector were measured by two complementary approaches. First, using live microscopy and a GFP-tagged version of the SipA chaperone, InvB, produced by a genetically engineered eukaryotic host cell, the secretion kinetics were determined by monitoring and quantifying InvB-GFP recruitment to contact sites between *Salmonella* and the cell. This elegant approach can not be applied to all effectors, since it requires the identification of a high-affinity partner (here the chaperone). As the second readout, the redistribution of SipA from the bacterial cytoplasm to its periphery was measured, on samples fixed at various times after live microscopy, from the moment of contact between the bacteria and the cell. Although this could in theory be automated to some extent, it requires very clean antibodies and extensive image acquisition. Another disadvantage was that detection of SipA with antibodies required sample fixation, preventing live imaging and analysis of later events.

In a different approach, two early effectors of *S. flexneri*, IpaB, and IpaC, were tagged with a tetracysteine tag. By loading the bacteria with fluorescent FlAsH probes, it was demonstrated that both effectors were secreted instantly after contact with host cells, with a half maximal rate of 4 min in both cases (Enninga et al., 2005). FlAsH labeling yields a somewhat poor signal-to-noise ratio, and is probably not appropriate for effectors of moderate or low abundance. The signal was only poorly detectable in the host cell, and loss of effector from the bacteria was recorded.

One current limitation of all the live imaging techniques is that they allow tracking of only one effector at a time. In order to gain insight into the hierarchy of secretion of different effectors, it is therefore necessary to compare the kinetics of secretion measured separately. For instance, the rate of secretion of tetracysteine-tagged SopE2 was found to be about 2-fold faster than that of SptP, another *Salmonella* effector, explaining how two effectors with antagonistic effects on the host cell could cooperate during the infectious process (Van Engelenburg and Palmer, 2008).

**Figure 5:**
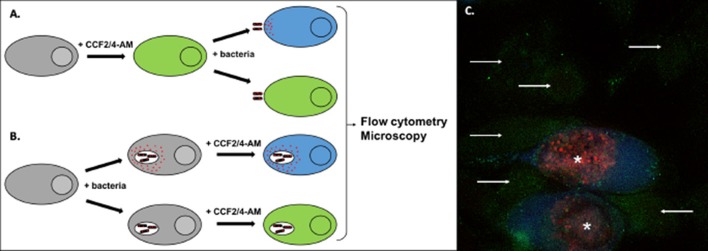
Detection of effector translocation using the beta-lactamase/CCF2/4-AM system. **(A,B)** Schematic view of the experimental set-up to test the translocation of a bacterial protein fused to beta-lactamase (red dots). To monitor secretion from extracellular bacteria, or from intracellular bacteria at an early stage of infection, cells are usually pre-loaded with the CCF2/4-AM **(A)**. Alternatively, the probe can be added to cells after infection, to monitor secretion events that occur later in infection **(B)**. Conversion of the fluorescent probe can be measured by microscopy or by flow cytometry. **(C)** Illustration of the analysis using microscopy. HeLa cells were infected for 40 h with *Chlamydia trachomatis* stably transformed with a plasmid expressing the translocated protein TarP in fusion with beta-lactamase and mCherry (Mueller and Fields, 2015). In the last 2 h of incubation the cells were loaded with CCF4-AM probe, before fixation. In uninfected cells the probe emits green fluorescence (arrows). In infected cells (bacteria in red, asterisks) translocation of the beta-lactamase activity into the cytosol is revealed by the appearance of a blue fluorescent signal, corresponding to the cleaved probe.

Currently, the most widely used assay is based on the development of fluorogenic substrates of beta-lactamase, and has been extensively applied to monitor secretion in T3SS and T4SS. Effector proteins are fused to beta-lactamase, while host cells are pre-loaded with the membrane-permeable substrate coumarin cephalosporin fluorescein (CCF2 or CCF4). The injection of the effector/beta-lactamase fusion protein into the host cytosol is detected by the loss of FRET upon cleavage of the fluorogenic substrate, inducing a switch in the fluorescence from green to blue (Charpentier and Oswald, 2004; Zuverink and Barbieri, 2015; Figure 5). This system has for instance been used with the beta-lactamase reporter encoded chromosomally in fusion to twenty different enteropathogenic *E. coli* (EPEC) effectors (Mills et al., 2013). Changes in the fluorescence of the CCF2 were monitored at 90 s intervals, and the secretion kinetics of ten different effectors was determined through this approach.

Although less resolutive in time, measurements on whole populations can provide information as to the order of effector secretion under some circumstances. *Chlamydia trachomatis* secretes several effectors, e.g., TarP and TepP, upon contact with the host cell. Once in the cytosol, TarP and TepP are tyrosine phosphorylated by host kinases. TarP phosphorylation was detectable as early as 5 min post infection whereas TepP phosphorylation occurred only between 5 and 15 min post infection, indicating that TarP injection occurs first (Chen et al., 2014). A similar strategy, combined with the addition of tags that become phosphorylated in the eukaryotic environment (Day et al., 2003; Garcia et al., 2006), could, in theory, be applied to several effectors, provided that they differ in size. Using an identical tag for each effector would limit the possibility that a difference in phosphorylation rates only reflects substrate preference by the host kinases involved.

On a larger scale, bio-orthogonal non-canonical amino acid tagging (BONCAT), combined with pulse labeling of the methionine surrogate, introduced the possibility to track the injection of endogenous proteins. Using this elegant strategy, Mahdavi and collaborators were able to characterize the order and pace of secretion, over 3 h of infection, of 11 Yop effectors from *Yersinia enterocolitica* (Mahdavi et al., 2014).

### When does secretion occur in a complex environment?

Although the complexity of secretion has been explored experimentally *in vitro* at a single cell level, fewer studies have tried to unravel the secretion complexity in more complex systems such as a population of cells and bacteria or even during infection in animal models.

The heterogeneity in secretion rates between different effectors led Mills and collaborators to pay attention not only to effectors but also to the target cells. By single cell tracking in cultures loaded with CCF2 and infected with either *Salmonella* Typhimurium or EPEC producing effectors fused to beta-lactamase, they identified a population of cells that were resistant to secretion, for which, despite the contact with bacteria, the fluorescence of the CCF2 remained unchanged even a couple of hours after infection. Whether this resistance to secretion is conferred by the host cell or is due to heterogeneities in the bacterial population remains unclear (Mills et al., 2013).

Only few studies have analyzed bacterial protein secretion in whole organisms. One example is the study of the activation of T3SS in whole plants, as was mentioned above (Monteiro et al., 2012). The early production of the T3S effector ExoU of *P. aeruginosa* and its secretion were shown to be critical for the development of the pathology in the lung of infected mice, using inducible production of the protein and immunolabelling on histological sections with a specific antibody to ExoU (Howell et al., 2013).

In a more complex system, Rolán and collaborators tackled the challenge of identifying the targets of the *Yersinia* effector YopH *in vivo* in mice, the main difficulty being the isolation of the few neutrophils targeted by the bacteria. In order to achieve this, they fused the beta-lactamase reporter to the first 100 amino acids of YopE. The splenocytes were then purified and loaded with CCF4 *ex vivo*, and the neutrophils injected with YopE were sorted from non-targeted cells on the basis of their fluorescence profile. This strategy allowed the authors to identify YopH-targeted signal transduction pathways that impair neutrophil responses *in vivo* (Hortensia Rolán et al., 2013).

Very recently, a genome-wide method, named EXIT (exported *in vivo* technology), was developed to identify proteins that are exported by bacteria during infection (Perkowski et al., 2017). EXIT utilizes the TEM beta-lactamase reporter lacking its native signal peptide, which, when fused in-frame to an export signal (i.e., signal peptide or transmembrane domain) confers beta-lactam resistance. By combining a comprehensive library of in-frame TEM fusions with the ability to select bacteria exporting fusion proteins *in vivo* and next-generation sequencing en masse of the recovered fusions, EXIT identified 593 proteins exported by *Mycobacterium tuberculosis* during infection in mice (Perkowski et al., 2017). Fifty-seven percent of these hits were predicted integral membrane proteins and 38% contained a predicted signal peptide. EXIT also identified 32 proteins (5%) lacking *in silico* predicted export signals.

## Concluding remarks

In the last decade, the repertoires of bacteria known to secrete proteins and of secreted proteins have expanded, and several novel secretion machineries have been discovered. These findings were mostly driven by technological advances, such as the discovery of secretion substrates through phage display coupled to next generation sequencing or through proteomics, independently of the secretion mechanism used, or by the development of probes that allowed sensitive detection of effector injection into a neighboring cell. Machine learning approaches have also pointed to new potential secretion substrates, and, with the expansion of the training sets (i.e., validated substrates and characterized secretion machineries) these approaches will likely be applicable to more secretion systems. In parallel, remarkable progress has been made in the exploration of the structure of the multicomponent secretion machineries using cryo-electron microscopy and tomography that should allow us in the future to understand their molecular mechanisms and their dynamic behavior. Still, very fundamental questions regarding the specificity of these machines remain unanswered. In many cases, the nature of the signal(s) that designate a protein as a secretion substrate and its recognition by the given machinery are still poorly understood. Insights into substrate recognition require detailed structural knowledge of a dynamic process that likely involves a series of intermediates. Capturing these intermediates has been extremely challenging, and their structure was resolved in part only in the Sec system. Cross-linking or mutagenesis approaches might enable one to stabilize and study these intermediate states. Computational approaches, including structural modeling and molecular dynamics have the potential to predict the details of these transient interactions and, combined with other validation tools, improve our understanding of transport processes. Finally, recent developments in high-resolution microscopy and tomography have provided important information on the architecture of secretion systems *in situ*, as a valuable basis for future studies (Chang et al., 2016; Hu et al., 2017). More studies are needed to understand how the secretion of different substrates by the same machinery is regulated. The design of probes that would allow one to track the secretion of two proteins simultaneously in living cells would certainly also help in addressing this question. Some of the tools described in this review are amenable to single-cell studies, and might reveal heterogeneity in the secretory behavior of bacteria, which has not been addressed so far. However, the next main challenge seems to be to probe bacterial secretion in complex environments such as biofilms, mixed microbial populations or within living hosts. Only a few of the approaches described here can be used in complex environments: our toolbox needs yet more new tools.

## Author contributions

BM, OF, and AS conceived and wrote the manuscript.

### Conflict of interest statement

The authors declare that the research was conducted in the absence of any commercial or financial relationships that could be construed as a potential conflict of interest.
